# Engineering de novo anthocyanin production in *Saccharomyces cerevisiae*

**DOI:** 10.1186/s12934-018-0951-6

**Published:** 2018-07-03

**Authors:** Mark Levisson, Constantinos Patinios, Sascha Hein, Philip A. de Groot, Jean-Marc Daran, Robert D. Hall, Stefan Martens, Jules Beekwilder

**Affiliations:** 1 Laboratory of Plant Physiology, Wageningen University & Research, Droevendaalsesteeg 1, 6708 PB Wageningen, The Netherlands; 2 Department of Food Quality and Nutrition, Fondazione Edmund Mach, Centro Ricerca e Innovazione, Via E. Mach, 1, 38010 San Michele all’Adige, TN Italy; 3 Department of Biotechnology, Delft University of Technology, van der Maasweg 9, 2629 HZ Delft, The Netherlands; 4 Wageningen Plant Research, Wageningen University & Research, Droevendaalsesteeg 1, 6708 PB Wageningen, The Netherlands

**Keywords:** Metabolic engineering, *Saccharomyces cerevisiae*, Natural products, Plant secondary metabolites, Pelargonidin, Flavonoids, Anthocyanin

## Abstract

**Background:**

Anthocyanins are polyphenolic pigments which provide pink to blue colours in fruits and flowers. There is an increasing demand for anthocyanins, as food colorants and as health-promoting substances. Plant production of anthocyanins is often seasonal and cannot always meet demand due to low productivity and the complexity of the plant extracts. Therefore, a system of on-demand supply is useful. While a number of other (simpler) plant polyphenols have been successfully produced in the yeast *Saccharomyces cerevisiae*, production of anthocyanins has not yet been reported.

**Results:**

*Saccharomyces cerevisiae* was engineered to produce pelargonidin 3-*O*-glucoside starting from glucose. Specific anthocyanin biosynthetic genes from *Arabidopsis thaliana* and *Gerbera hybrida* were introduced in a *S. cerevisiae* strain producing naringenin, the flavonoid precursor of anthocyanins. Upon culturing, pelargonidin and its 3-*O*-glucoside were detected inside the yeast cells, albeit at low concentrations. A number of related intermediates and side-products were much more abundant and were secreted into the culture medium. To optimize titers of pelargonidin 3-*O*-glucoside further, biosynthetic genes were stably integrated into the yeast genome, and formation of a major side-product, phloretic acid, was prevented by engineering the yeast chassis. Further engineering, by removing two glucosidases which are known to degrade pelargonidin 3-*O*-glucoside, did not result in higher yields of glycosylated pelargonidin. In aerated, pH controlled batch reactors, intracellular pelargonidin accumulation reached 0.01 µmol/g_CDW_, while kaempferol and dihydrokaempferol were effectively exported to reach extracellular concentration of 20 µM [5 mg/L] and 150 µM [44 mg/L], respectively.

**Conclusion:**

The results reported in this study demonstrate the proof-of-concept that *S. cerevisiae* is capable of de novo production of the anthocyanin pelargonidin 3-*O*-glucoside. Furthermore, while current conversion efficiencies are low, a number of clear bottlenecks have already been identified which, when overcome, have huge potential to enhance anthocyanin production efficiency. These results bode very well for the development of fermentation-based production systems for specific and individual anthocyanin molecules. Such systems have both great scientific value for identifying and characterising anthocyanin decorating enzymes as well as significant commercial potential for the production of, on-demand, pure bioactive compounds to be used in the food, health and even pharma industries.

**Electronic supplementary material:**

The online version of this article (10.1186/s12934-018-0951-6) contains supplementary material, which is available to authorized users.

## Background

Anthocyanins are water-soluble pigments that colour the leaves, fruit and flowers of many plant species. Despite the small number of aglycones (anthocyanidins) produced by the plant kingdom, very many different anthocyanins have been identified and distinguished by their unique degree of decoration achieved by methylation, glycosylation and acylation with both aliphatic and aromatic groups. These decorations convey a contrasting set of characteristics to the molecules (stability, solubility, bioavailability, anti-oxidant properties, etc.) and have direct relevance to their importance in a food/health context. In our diet, anthocyanins occur as pigments [1], but anthocyanins and their derivatives are increasingly being considered as interesting bioactive compounds with various health promoting effects [2]. This is supported by many more recent studies of anthocyanin-rich fruits, such as blueberry and cranberry [3]. Their relative abundance in the diet and their potency against a range of chronic diseases have made anthocyanins the subject of intense research in experimental and preventive medicine. Furthermore, more recently there is a fast-growing market demand for formulating natural colours [4]. The limited range of commercially-available anthocyanins and the expense of making pure preparations means that most research has been done only using crude extracts of plants. These plant-derived anthocyanin extracts are typically not standardised with respect to the particular anthocyanins they contain, nor to the amounts of each anthocyanin in the extract. Variations in anthocyanin decoration account for differences in colour stability and pigment hue and this underpins the growing need for developing systems for the production of pure anthocyanins, for investigating the effects of chemical specificity on uptake, signalling and physiology, toxicity of anthocyanins for medical applications and for developing new formulations for the food pigment industries [5].

**Fig. 1:**
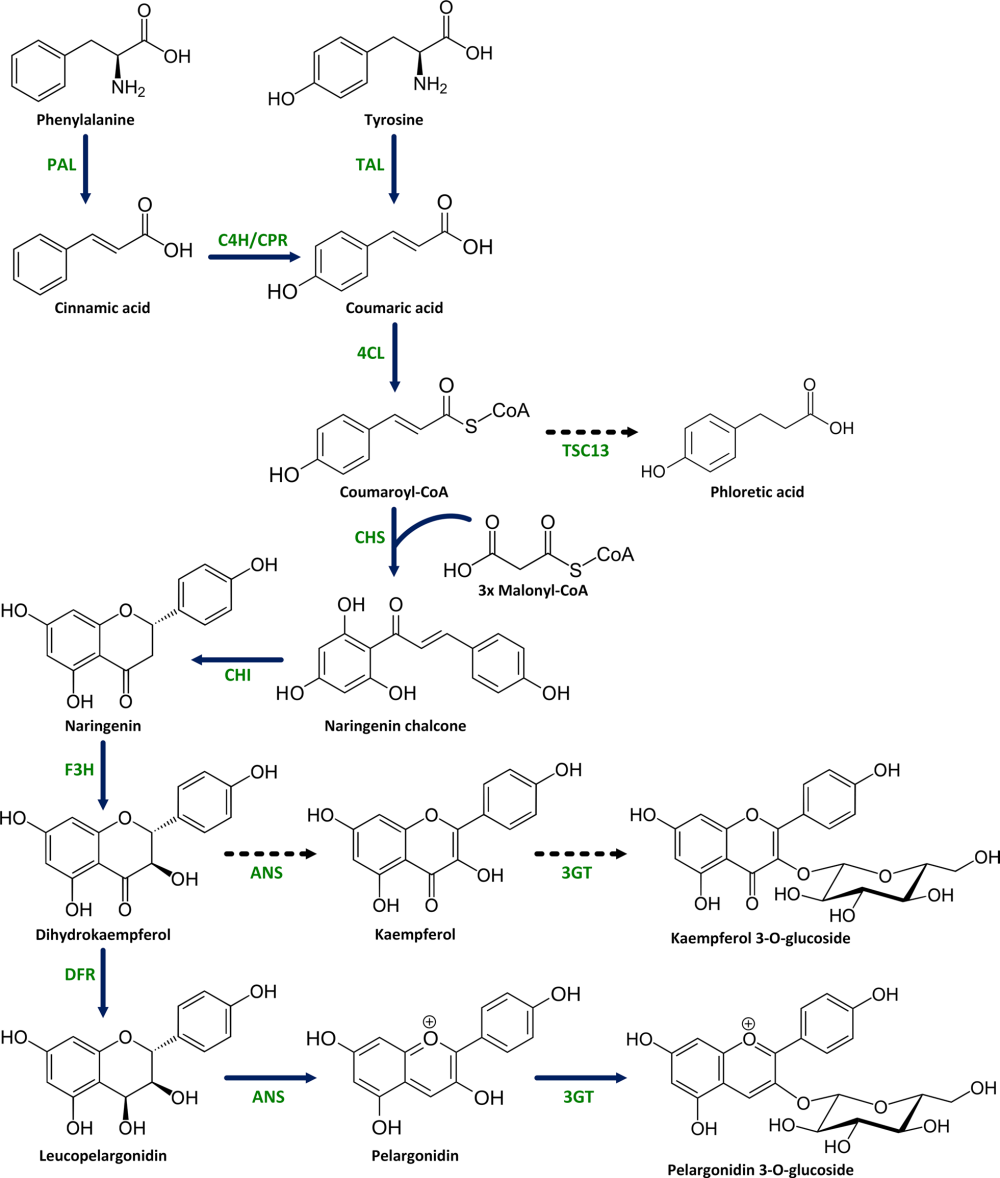
Representation of the integrated pelargonidin 3-*O*-glucoside biosynthesis pathway. Nine *A. thaliana* genes were overexpressed: *PAL* phenylalanine ammonia lyase, *C4H* cinnamate 4-hydroxylase, *CPR* cytochrome P450 reductase, *4CL* 4-coumaric acid-CoA ligase, *CHS* chalcone synthase, *CHI* chalcone isomerase, *F3H* flavanone 3-hydroxylase, *ANS* anthocyanidin synthase, and 3GT anthocyanin 3-*O*-glucosyltransferase; one gene from *G. hybrida*: *DFR* dihydroflavonol 4-reductase; and one gene from *R. capsulatus*: *TAL* tyrosine ammonia lyase. Enzyme names are in green. Bold dark blue arrows indicate the pelargonidin 3-*O*-glucoside biosynthesis pathway. Dashed lines indicate routes to side-products produced by the catalytic activity of ANS, 3GT or the endogenous TSC13 (a very long chain fatty acid enoyl reductase)

The anthocyanin pathway is one of best understood and characterized pathways in plant secondary metabolism both in genetic as in chemical/enzymatic terms [6]. It branches from the flavonoid pathway after the formation of flavanones, such as naringenin and eriodictyol (Fig. 1). The availability of genes for the entire pathway opens up the possibility for the expression of the full plant biosynthetic pathway in a microbial host and hence create a sustainable tailor-made platform for the biotechnological production of specific anthocyanin derivatives. Naringenin is synthesized via the phenylpropanoid pathway, starting from the aromatic amino acids phenylalanine or tyrosine. Yeast strains engineered with the pathway towards naringenin have been described [7, 8]. Starting from naringenin, anthocyanidins are biosynthesized by flavanone 3-hydroxylase (F3H; syn. FHT), dihydroflavonol 4-reductase (DFR), and anthocyanidin synthase (ANS; syn. leucoanthocyanidin dioxygenase). ANS has been characterized as a multifunctional protein catalysing several reactions with different flavonoid substrate intermediates [9, 10]. The final step towards a basic anthocyanin is catalysed by an anthocyanin 3-*O*-glucosyltransferase (3GT). Other modifications of the anthocyanidin skeleton can be introduced at different points in this pathway, for instance by the action of flavonoid hydroxylases, methyltransferases, other glycosyltransferases and acyltransferases [11].

Anthocyanin production has been achieved in *Escherichia coli*, by introducing parts of the anthocyanin biosynthetic pathway and by supplementing the growth medium with precursors such as coumaric acid, naringenin, catechin, or eriodyctiol [12–16]. Several strategies have been developed to improve further the anthocyanin yield in this system, including a higher availability of UDP-glucose, redirecting the carbon metabolism to malonyl-CoA and optimizing cultivation parameters. These led to the production of the anthocyanin cyanidin 3-*O*-glucoside (C3G) at a titer of 350 mg/L [15]. Recently, a four-strain *E. coli* polyculture was used to obtain de novo production of pelargonidin 3-*O*-glucoside at a titer of 9.5 mg/L [17]. However, despite improvements at the genetic and cultivation levels, the construction of a single microbial strain capable of producing de novo anthocyanins and utilizing a simple carbon source has not been reported.

In a previous work, we developed strains of the yeast *S. cerevisiae* that were capable of producing 400 µM naringenin in their culture medium [7]. In the present study, this platform has been expanded towards the production of the anthocyanidin pelargonidin and its glucoside, pelargonidin 3-*O*-glucoside. A number of limitations of the established system were revealed by the detection of side-products in the cultures, and attempted solutions to these limitations are discussed.

## Results

### Selection of genes necessary for the synthesis of anthocyanins in *S. cerevisiae*

In previous work, the *S. cerevisiae* strain IMX106 had been engineered to produce naringenin and was reported to produce up to 46.5 µM of the flavonoid [7]. In order to convert naringenin into the anthocyanin pelargonidin 3-*O*-glucoside, four additional enzymatic steps are required (Fig. 1). To match the gene origin used to convert aromatic amino acids to naringenin in strain IMX106, which still has a number of auxotrophic markers available, the *F3H*, *ANS* and *UGT78D2* (*3GT*) genes for anthocyanin biosynthesis were all, but *DFR*, recruited from *A. thaliana*. The *A. thaliana DFR* gene was not used because it is known only to accept dihydroquercetin (DHQ) and dihydromyricetin (DHM) as substrates and is unable to convert the expected substrate in this system, dihydrokaempferol (DHK) into leucopelargonidin [14, 18] (Fig. 1). In contrast, the DFR gene from *G. hybrida* has been shown to use DHK and was, therefore, selected instead to complete the biosynthetic pathway [19]. Episomal expression vectors, carrying combinations of yeast codon-optimized versions of the *A. thaliana F3H* (*coF3H*), *ANS* (*coANS*), *UGT78D2* (*co3GT*) and *G. hybrida DFR* (*coGhDFR*), were constructed.

The episomal expression vectors pF and pFD were constructed to analyse the first two steps of anthocyanin biosynthesis. These plasmids were transformed into the naringenin-producing strain IMX106, resulting in strains IMX106-F and IMX106-FD. These strains were cultured in shake-flasks with 10 mL synthetic medium supplemented with glucose as sole carbon source for 65 h, after which the total culture was extracted, and extracts analysed using HPLC. Strain IMX106-F was able to efficiently produce DHK from naringenin (Table 1). Production of DHK by strain IMX106-FD was comparable to strain IMX106-F, suggesting that DFR activity was very low. Nevertheless, production of leucoanthocyanidin could be detected via a colorimetric assay (see Additional file 1). To initiate the conversion of leucopelargonidin to pelargonidin the *coANS* gene was inserted into the pFD plasmid. The resulting strain IMX106-FDA showed to produce, not only pelargonidin, but also high amounts of the side product kaempferol (Table 1). The ratio between pelargonidin and kaempferol concentrations reached a value of 6.10^−3^ (Table 1). To improve the stability of pelargonidin, the *co3GT* gene encoding a glucosyltransferase was incorporated into the pFDA plasmid. The resulting strain IMX106-FDA3 was able to produce small amounts of pelargonidin 3-*O*-glucoside (Table 1). However, a second major side product was also detected and subsequently identified as kaempferol 3-*O*-glucoside (K3G). Approximately 30% of the produced kaempferol was glycosylated by 3GT, while only 10% of the produced pelargonidin was found to be glycosylated. Although several conversions in this process apparently occur at suboptimal efficiency, this experiment nevertheless showed that de novo P3G production is indeed possible in *S. cerevisiae*.

**Table 1:** Product formation of naringenin and downstream metabolites in *S. cerevisiae* strains.

µM	IMX106	IMX106-F	IMX106-FD	IMX106-FDA	IMX106-FDA3
Naringenin	23.4 ± 1.4	7.8 ± 0.1	7.7 ± 0.6	6.5 ± 0.3	3.1 ± 1.0
DHK	–	18.5 ± 0.1	17.5 ± 0.9	8.5 ± 0.00	9.8 ± 0.8
Kaempferol	–	–	–	11.7 ± 0.5	4.4 ± 1.0
K3G	–	–	–	–	2.3 ± 0.2
Pelargonidin	–	–	–	0.07 ± 0.00	0.85 ± 0.03
P3G	–	–	–	–	0.05 ± 0.00

Strains IMX106, IMX106-F, IMX106-FD, IMX106-FDA and IMX106-FDA3 were grown in 10 mL shake-flask cultures. The whole culture was extracted after 65 h of culturing at 30 °C. Metabolite concentrations of naringenin, dihydrokaempferol (DHK), kaempferol, kaempferol 3-*O*-glucoside (K3G), pelargonidin and pelargonidin 3-*O*-glucoside (P3G) expressed in µM were measured by HPLC. Data represents the average ± mean deviation of independent biological triplicates

– not detected

#### Effect of different DFRs on P3G production

**Fig. 2:**
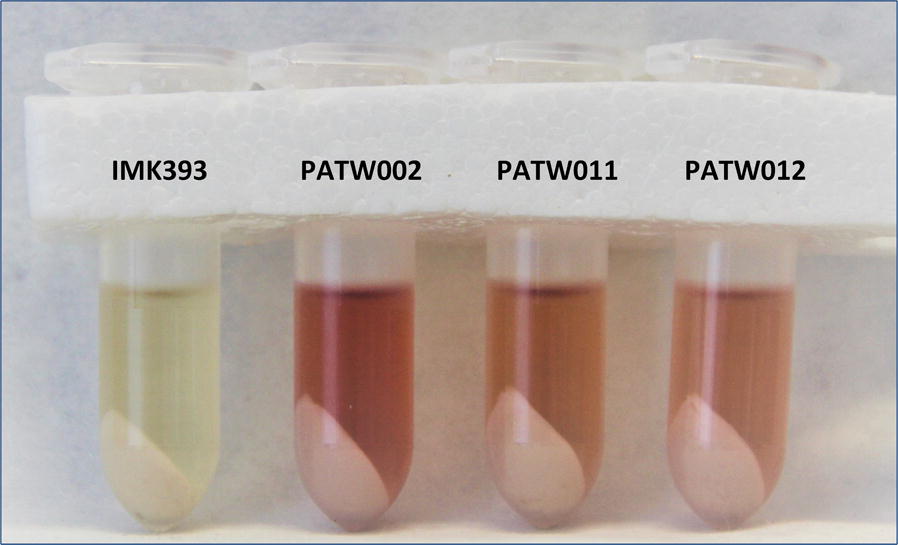
Extracts of cell pellets from *S. cerevisiae* strains PATW002, PATW011 and PATW012. These strains express *coGhDFR*, *coMtDFR1* and *coAaDFR* respectively in combination with *coF3H*, *coANS*, *co3GT* genes. The strains were grown in SMNar (1.5 mM naringenin) and the cell pellets were lyophilized and extracted with acidified methanol

From the massive accumulation of DHK in the previous experiment it was evident that DFR activity is one of the bottlenecks in P3G production. Therefore, to engineer efficient P3G biosynthesis in *S. cerevisiae*, we then selected and tested two additional yeast codon-optimized DFR enzymes (from *Medicago truncatula* and *Anthurium andreanum*) in addition to the DFR from *G. hybrida*, each of which had previously been shown to reduce DHK to leucopelargonidin [14, 18–20]. The episomal expression vectors pCPW002, pCPW006 and pCPW007 were constructed containing the *coF3H*, *coANS*, *co3GT* and the *coGhDFR*, *coMtDFR* or *coAaDFR* genes, respectively. These plasmids were transformed to strain IMK393 (*aro3Δ*, *ARO4*^*G226S*^, *pdc6Δ*, *pdc5Δ*, *aro10Δ*), resulting in strains PATW002, PATW011, and PATW012. These strains were cultured in shake-flasks with SMNar (1.5 mM naringenin) and glucose as sole carbon source for 140 h. HPLC analysis of culture supernatant showed the production of DHK, while pelargonidin or P3G were not detected (Table 2). On the other hand, the two by-products, kaempferol and kaempferol 3-*O*-glucoside, were detected in the culture supernatant. At the end of the 140 h, biomass was harvested and yeast cell pellets were extracted (Fig. 2) and analysed for intracellular pelargonidin and P3G using HPLC and LC–MS. The strain PATW002, expressing *coGhDFR*, produced twice the amount of pelargonidin relative to strains PATW011 and 012 (Table 2). Again, only traces of P3G could be detected, using LC–MS.

**Table 2:** Product formation in *S. cerevisiae* strains PATW002, PATW011 and PATW012 in shake flask cultures

	PATW002	PATW011	PATW012	PATW002	PATW011	PATW012
	[Concentration]_Ext_ µM			[Concentration]_Int_ µmol/g_CDW_		
DHK	60.7 ± 2.3	77.0 ± 3.1	43.3 ± 0.7	2.25 ± 0.17	1.64 ± 0.18	0.84 ± 0.04
Kaempferol	2.7 ± 0.2	1.5 ± 0.2	5.0 ± 0.2	1.76 ± 0.07	0.55 ± 0.04	1.45 ± 0.05
K3G	50.3 ± 3.6	12.4 ± 0.3	33.3 ± 1.9	1.58 ± 0.14	0.26 ± 0.02	0.49 ± 0.05
Pelargonidin	–	–	–	0.066 ± 0.01	0.019 ± 0.00	0.026 ± 0.00
P3G	–	–	–	–	–	–

These strains express *coGhDFR*, *coMtDFR1* and *coAaDFR,* respectively in combination with *coF3H, coANS, co3GT* genes. The strains were grown in shake-flasks with 50 mL SMNar (1.5 mM naringenin) and extracellular metabolite concentration of dihydrokaempferol (DHK), kaempferol, kaempferol 3-*O*-glucoside (K3G), pelargonidin and pelargonidin 3-*O*-glucoside (P3G) expressed in µM were measured by HPLC in supernatant sampled after 140 h of cultures at 30 °C. Growth and production time courses of extracellular metabolites can be found in Additional file 2. Intracellular concentrations expressed in µmol/g_CDW_ were extracted and measured by HPLC. Data represent the average ± mean deviation of independent biological triplicates

– not detected

Having established a genetic blueprint necessary for de novo synthesis of anthocyanins in *S. cerevisiae*, the next step was to express the selected gene set in an optimized metabolic chassis.

### Optimization of a *S. cerevisiae* chassis for anthocyanin synthesis

#### Integration of the anthocyanin biosynthetic pathway in the yeast genome

Previously, *S. cerevisiae* was engineered for de novo naringenin production by introducing the naringenin biosynthetic genes from *A. thaliana* and optimizing the flux through the shikimate pathway [7]. This engineered yeast (IMX198) for naringenin production provides the metabolic chassis for the current study. This strain, however, relies on plasmid-based overexpression of genes. Direct integration of genes is more stable and reliable compared with plasmid-based overexpression. Therefore, as a first step, a strain was created in which the biosynthetic genes towards naringenin (*PAL*, *C4H*, *CPR*, *4CL*, *CHS* and *CHI*) were integrated into the genome of *S. cerevisiae* strain IMK393, which carries a set a mutations resulting in deregulation of the aromatic amino acid biosynthetic pathway and elimination of the Ehrlich pathway. Two separate genomic locations (X-2 and XII-2) were selected to integrate the naringenin biosynthetic genes. Both locations have been previously validated for stable integration of multiple genes and strain fitness [21]. First, the *atCHI1*, *atCHS3* and *at4CL3* genes were integrated at the X-2 integration site of strain IMK393, resulting in strain PATW004. Subsequently, the *atPAL1*, *coC4H*, *coCPR1*, *and coCHS3* genes were integrated into the XII-2 integration site of PATW004, resulting in strain PATW066. The newly constructed naringenin-producing strain PATW066 (*aro3Δ*, *ARO4*^*G226S*^, *pdc6Δ*, *pdc5Δ*, *aro10Δ*, *atPAL1↑*, *coC4H↑*, *coCPR1↑*, *atCHI1↑*, *atCHS3↑*, *coCHS3↑*, *at4CL3↑*) exhibited flavonoid production characteristics very similar to the IMX106 strain that was used to perform the anthocyanin genes selection (Table 3). HPLC analysis of the culture supernatant showed naringenin production up to a concentration of 40 µM and production of phloretic acid up to 160 µM for both strains (Table 3). Thus strain PATW066 was used as the new naringenin-producing platform. In a second step, the genes *coF3H*, *coGhDFR*, *coANS* and *co3GT* were introduced in PATW066 by integration at the *CAN1* locus, resulting in strain PATW079. The *CAN1* locus is an endogenous genomic negative selectable marker that is commonly used for integrations [22, 23]. The resulting strain PATW079 (*aro3Δ*, *ARO4*^*G226S*^, *pdc6Δ*, *pdc5Δ*, *aro10Δ*, *can1Δ, atPAL1↑*, *coC4H↑*, *coCPR1↑*, *atCHI1↑*, *atCHS3↑*, *coCHS3↑*, *at4CL3↑*, *coF3H↑*, *coGhDFR↑*, *coANS↑*, *co3GT↑*) was cultured in shake-flasks on SM medium with glucose as sole carbon source for 144 h. HPLC analysis of the culture supernatant and cell pellet showed the production of naringenin, phloretic acid, coumaric acid, as well as DHK, kaempferol, K3G and pelargonidin (Table 3).

**Table 3:** Product formation in *S. cerevisiae* strains PATW066, PATW079, PATW080 and PATW076 in shake flask cultures

	IMX106^a^	PATW066	PATW079	PATW080	PATW076	PATW079	PATW080	PATW076
	[Concentration]_Ext_ µM					[Concentration]_Int_ µmol/g_CDW_		
Coumaric acid	25	2.4 ± 0	2.8 ± 0	21.2 ± 9	51.6 ± 5	0.02 ± 0.00	0.24 ± 0.01	0.50 ± 0.02
Phloretic acid	200	167.2 ± 7	200.2 ± 2	139.5 ± 8	–	–	–	–
Naringenin	46.5	40.0 ± 4	1.8 ± 0	1.9 ± 0	3.6 ± 0	0.11 ± 0.07	0.15 ± 0.01	0.32 ± 0.02
DHK	^–^	–	28.8 ± 2	33.2 ± 3	92.5 ± 4	0.45 ± 0.04	0.71 ± 0.06	1.71 ± 0.12
Kaempferol	–	–	–	0.3 ± 0	0.6 ± 0	0.11 ± 0.01	0.17 ± 0.03	0.36 ± 0.03
K3G	–	–	8.3 ± 1	7.5 ± 1	16.1 ± 1	0.09 ± 0.03	0.14 ± 0.02	0.22 ± 0.01
Pelargonidin	–	–	–	–	–	0.003 ± 0.001	0.005 ± 0.001	0.011 ± 0.002
P3G	–	–	–	–	–	–	–	–

The strains were grown in shake-flasks with 50 mL SMG and extracellular metabolite concentration of coumaric and phloretic acids, naringenin, dihydrokaempferol (DHK), kaempferol, kaempferol 3-*O*-glucoside (K3G), pelargonidin and pelargonidin 3-*O*-glucoside (P3G) expressed in µM were measured by HPLC in supernatant sampled after 140 h of cultures at 30 °C. Growth and production time courses of extracellular metabolites can be found in Additional file 3. Intracellular concentrations expressed µmol/g_CDW_ were extracted and measured by HPLC. Data represent the average ± mean deviation of independent biological triplicates

– not detected

^a^ Data from [7] (140 h of growth of IMX106)

#### Deletion of *EXG1* and *SPR1*, genes encoding β-glucosidases

The imbalance observed in pelargonidin and pelargonidin 3-*O*-glucoside might originate from a poor performance of the glucosyltransferase or to the presence of native glucosidase activity. We could undeniably confirm the activity of the 3-*O*-glycosyltransferase thanks to the detectable concentration of kaempferol 3-*O*-glucoside in the strain PATW079. This might then suggest that breakdown of pelargonidin 3-*O*-glucoside may occur in yeast, as previously proposed [24]. Several *S. cerevisiae* glucosidases have been identified that can hydrolyse flavonoid glucosides and convert them back to their aglycone form [24]. Significant hydrolysis of anthocyanins, including P3G, were catalysed by the translational product of the glucosidase genes *EXG1* and *SPR1*. In another study, it was shown that the production of the flavonoid glucoside scutellarein 7-*O*-glucoside was increased when these glucosidases were deleted [25]. Therefore, for our study, the *EXG1* and *SPR1* genes were deleted in the anthocyanin-producing strain PATW079. Each glucosidase was replaced by a gene that will contribute to naringenin production and should consequently lead to improved pelargonidin and pelargonidin 3-*O*-glucoside production. Previously, it was shown that the introduction of the tyrosine ammonia lyase (*coTAL1*) gene from *Rhodobacter capsulatus*, which converts tyrosine into coumaric acid (see Fig. 1), together with additional copies of *coCHS3*, improved naringenin production [7]. Therefore, the *EXG1* and *SPR1* knockouts were combined with the introduction of an expression cassette of codon optimized *coCHS3* and *coTAL1*, respectively, resulting in strain PATW080 (*aro3Δ*, *ARO4*^*G226S*^, *pdc6Δ*, *pdc5Δ*, *aro10Δ*, *can1Δ*, *exg1Δ*, *spr1Δ*, *atPAL1↑*, *coC4H↑*, *coCPR1↑*, *atCHI1↑*, *atCHS3↑*, *coCHS3↑*, *at4CL3↑*, *coF3H↑*, *coGhDFR↑*, *coANS↑*, *co3GT↑*, *coCHS3↑*, *coTAL1↑*). This latter strain was cultivated under shake flask conditions using SMG and HPLC analysis of culture supernatant and pellet showed that the levels of phloretic acid declined, likely due to the presence of an additional copy of *coCHS3* in PATW080, relative to PATW079, while increases were observed in the concentrations of DHK, K3G, kaempferol and pelargonidin (Table 3). Interestingly, deletion of *EXG1* and *SPR1* did not yield significant changes in measurable quantities of P3G and did not influence the ratio of kaempferol and K3G (Table 3).

#### Abolishing the competing pathway towards phloretic acid

The formation of phloretic acid as an unwanted by-product in flavonoid-producing yeast strains is associated with the presence of the 4-coumaric acid-CoA ligase (*4CL3*) gene, and leads to major losses of carbon entering the phenylpropanoid pathway [7, 26–28]. Recently, *S. cerevisiae TSC13* encoding a very long chain fatty acid (VLCFA) enoyl reductase was identified as the responsible enzyme for the formation of phloretic acid via the reduction of coumaroyl-CoA (Fig. 1) [29]. Replacing *TSC13* by *MdECR*, an ortholog from apple (*Malus domesticus*), was shown to eliminate phloretic acid production, while retaining good culture growth, and improved production of naringenin [30]. Therefore, the *TSC13* coding sequence in strain PATW080 was replaced with a yeast codon-optimized version of *MdECR* (*coMdECR*), which resulted in strain PATW076. PATW076 (*aro3Δ*, *ARO4*^*G226S*^, *pdc6Δ*, *pdc5Δ*, *aro10Δ*, *can1Δ*, *exg1Δ*, *spr1Δ*, *tsc13Δ*, *atPAL1↑*, *coC4H↑*, *coCPR1↑*, *atCHI1↑*, *atCHS3↑*, *coCHS3↑*, *at4CL3↑*, *coF3H↑*, *coGhDFR↑*, *coANS↑*, *co3GT↑*, *coCHS3↑*, *coTAL1↑*, *coMdECR*) was cultivated under shake-flask conditions using minimal medium and compared to PATW079 and PATW080. HPLC analysis of the culture supernatants and pellets showed that phloretic acid formation was indeed completely eliminated in PATW076, and the production of all other phenolic compounds, including coumaric acid, naringenin, dihydrokaempferol, kaempferol, kaempferol 3-*O*-glucoside and pelargonidin, was increased by twofold relative to PATW080 (Table 3).

### Anthocyanin production in controlled aerobic batch cultures

**Fig. 3:**
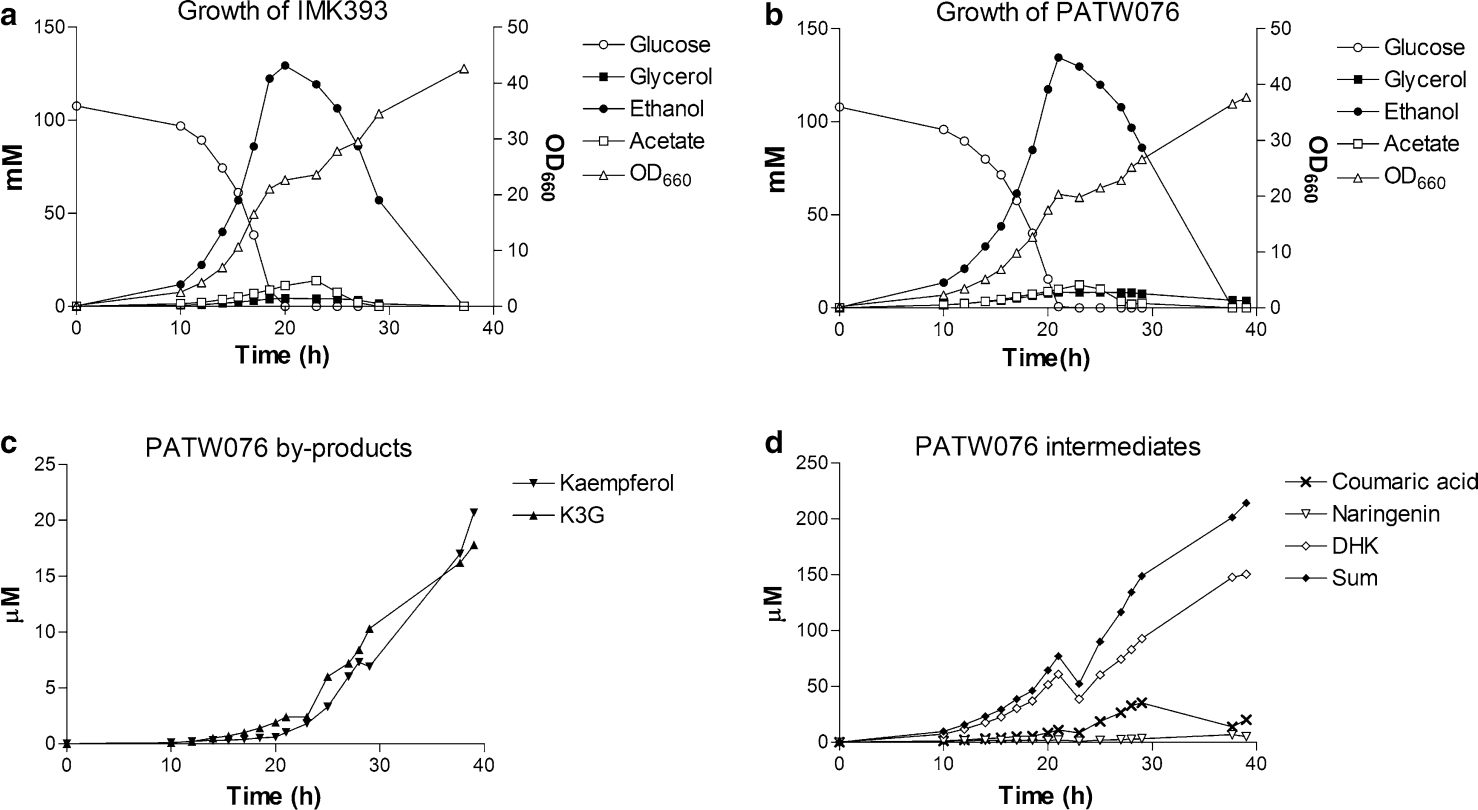
Growth of IMK393 & PATW076 and flavonoids formation by PATW076 in bioreactors. Growth and extracellular metabolite formation were studied in duplicate pH controlled (pH 5.0) and aerobic controlled batch cultures of IMK393 and PATW076 on glucose and auxotrophic supplements. **a** Concentrations of glucose (White circle), ethanol (Black circle), acetate (White square), glycerol (Black square), and optical density (OD_660_) (Triangle) for IMK393. **b** Concentrations of glucose (White circle), ethanol (Black circle), acetate (White square), glycerol (Black square), and optical density (OD_660_) (Triangle) for PATW076. **c** Concentrations of kaempferol (Black down-pointing triangle), and kaempferol 3-*O*-glucoside (K3G) (Black up-pointing triangle) for PATW076. **d** Concentrations of coumaric acid (Times), naringenin (Inverted triangle), dihydrokaempferol (White diamond), and the sum of all flavonoids (Black diamond) for PATW076. Results are shown from a single representative experiment

For further characterisation of strain PATW076 (*aro3Δ*, *ARO4*^*G226S*^, *pdc6Δ*, *pdc5Δ*, *aro10Δ*, *can1Δ*, *exg1Δ*, *spr1Δ*, *tsc13Δ*, *atPAL1↑*, *coC4H↑*, *coCPR1↑*, *atCHI1↑*, *atCHS3↑*, *coCHS3↑*, *at4CL3↑*, *coF3H↑*, *coGhDFR↑*, *coANS↑*, *co3GT↑*, *coCHS3↑*, *coTAL1↑*, *coMdECR*), this strain was grown in aerobic bioreactors operated as batch culture in SMG medium (20 g/L glucose) at pH 5.0. The strain IMK393 (*aro3Δ*, *ARO4*^*G226S*^, *pdc6Δ*, *pdc5Δ*, *aro10Δ*) was grown in the exact same conditions as a control (Fig. 3). In these aerobic cultures, glucose metabolism was predominantly fermentative producing ethanol, acetate, glycerol, CO_2_, and biomass, after which the metabolism switched towards reconsumption of ethanol, acetate, and glycerol (Fig. 3a, b) [31, 32]. The specific growth rates of the control strain IMK393 and the flavonoid producing PATW076 during the glucose consumption phase were 0.23 ± 0.02 and 0.18 ± 0.01/h, respectively. These specific growth rates were approximately half of specific growth rate from the reference strain CEN.PK113-7D, but are similar to the growth rate of the naringenin production strain IMX198 (described in [7]).

The total sum of extracellular flavonoids at the end of glucose consumption phase was only 70.4 ± 9.5 µM, consisting mostly of dihydrokaempferol with 59.9 ± 1.5 µM, whereas the concentration of flavonoids reached 202.3 ± 16.9 µM at the end of the fermentation (Fig. 3c, d). Although concentrations of intermediates like coumaric acid and naringenin decreased towards the end of the fermentation, similar to naringenin intermediates in Koopman et al. [7], the dihydrokaempferol concentration increased throughout the fermentation towards 151.8 ± 1.9 µM, indicating there is a limiting step towards anthocyanidin and anthocyanin production (Fig. 3d). Furthermore, the by-products kaempferol and kaempferol 3-*O*-glucoside were excreted to a final concentration of 18.3 ± 3.5 µM and 18.3 ± 1.9 µM (Fig. 3c).

Since pelargonidin and pelargonidin 3-*O*-glucoside could not be measured as extracellular metabolites in shake flask experiments, biomass samples were taken from the bioreactors after complete glucose consumption and after the reconsumption of ethanol for analysis of intracellular compounds in the anthocyanin pathway. Concentrations of intermediates coumaric acid, naringenin, and dihydrokaempferol roughly increased twice between the end of the glucose and ethanol growth phase, whereas concentrations for pelargonidin increased approximately four times from 0.002 ± 0.001 to 0.01 ± 0.002 µmol/g_CDW_ and five times for both kaempferol and kaempferol 3-*O*-glucoside with final concentrations of 0.96 ± 0.1 and 0.25 ± 0.01 µmol/g_CDW_. Pelargonidin 3-*O*-glucoside was estimated from LC–MS data to be 0.001 µmol/g_CDW_ (see Additional file 4).

## Discussion

In this work we provide a clear proof-of concept for the production of a complex plant polyphenol anthocyanin in *S. cerevisiae*. We describe the first example of de novo production of pelargonidin and pelargonidin 3-*O*-glucoside in baker’s yeast, starting from glucose. To establish the anthocyanin biosynthetic pathway in yeast, a previously-developed yeast strain with improved aromatic amino acid biosynthesis was further engineered by integrating fourteen additional genes into its genome. Moreover, three endogenous genes were deleted from the chassis genome to suppress by-product formation and degradation of the end-product. In an aerobic batch fermentation experiment, intracellular concentrations of 0.01 µmol/g_CDW_ pelargonidin could be achieved in these cultures.

In previous studies, the production of anthocyanin compounds was achieved in *E. coli* [33]. All work described in* E. coli* reports on bioconversion of an intermediate (e.g. naringenin), which was fed to the culture. Recently, the complete biosynthesis of anthocyanins starting from sugars using *E. coli* polycultures was described [17]. The biosynthesis of anthocyanins was distributed between several strains, the first producing an intermediate to feed another. This concept demonstrated to be superior to single strain engineering. However, the anthocyanin biosynthesis was not completely de novo since malonate was fed into the culture. Furthermore, in industrial biotechnology the use of polycultures still needs to be developed and will face additional challenges, while processes using monocultures are standard. In comparison to yeast, *E. coli* is not a food-grade microorganism and may have limitations in expressing cytochrome P450 enzymes for further modifications of anthocyanins. Production of anthocyanins by plant cell cultures has also been developed [34]. Plant cells in general produce complex mixtures of compounds, and are difficult to stabilize and engineer. Therefore, we aimed to design a single yeast strain capable of producing de novo anthocyanins and utilizing a simple carbon source. During review of this manuscript, production of anthocyanins in yeast using genes from a wide array of plants was published, which further reinforces our observations and complements the present study [35].

In previous studies, the de novo production of plant polyphenols, such as the flavanone naringenin, the stilbene resveratrol and the flavonol kaempferol was reported [7, 36, 37]. These achievements were ground-breaking to demonstrate that it was feasible to produce complex high-value phenolic compounds in yeast culture supernatants in a relatively simple fermentative set up. In the current study, we aimed to extrapolate these findings further towards anthocyanins, which exhibit a broad range of applications in the food, cosmetics and health sectors. Therefore it is an important observation that we can produce such anthocyanins in a fermentation system, albeit at levels that are as yet suboptimal and lower than their precursor molecule naringenin.

In spite several attempts to improve the synthesis of pelargonidin and its glycosylated form, including positive elimination of phloretic acid formation and deletion of endogenous glucosidases, it was obvious that many metabolic bottlenecks still occur and limit yet exploitation of these strains. Overcoming these current limitations has huge potential to increase overall end product yield. A major bottleneck in the production of anthocyanins is located in the steps after the formation of dihydrokaempferol from naringenin. It is clear that the majority of the naringenin formed is converted to dihydrokaempferol, indicating that F3H is functioning well (Tables 2 and 3). However, the enzymes responsible for the subsequent steps towards anthocyanin, DFR and ANS, clearly function less efficiently in the pathway.

The most obvious candidate for improvement is the DFR enzyme. A limited number of DFR enzymes from different plants have been tested, and only some of them were capable of converting dihydrokaempferol to leucopelargonidin. When comparing the efficiency of three of these DFRs in *S. cerevisiae*, only minor differences in (low) efficiency were observed (Table 2), indicating that the DFR reaction is not performing well in yeast. Similar observations have been made before, in prokaryotic hosts such as *E. coli* [38]. The background to this apparent malfunctioning of DFRs in microbial systems could relate to the exceptional level of substrate inhibition known to be characteristic for this enzyme. Trabelsi et al. have described a detailed study on kinetic parameters of the DFR enzyme from *Vitis vinifera* (*Vv*DFR), and have observed that high concentrations of substrate (dihydroquercetin in the case of *Vv*DFR) lead to the formation of dead-end enzyme–substrate complexes [39, 40]. Indeed, in our system we observe high concentrations of dihydrokaempferol. Therefore, regulating the expression of the F3H enzyme may be necessary to achieve optimal concentrations of dihydrokaempferol in order for DFR to function effectively without inhibition. Another explanation could be the findings of Halbwirth et al. that, in the presence of excess amounts of leucoanthocyanidin, DFR might catalyse the reverse reaction to form dihydroflavonols from leucoanthocyanidins [41, 42].

The ANS enzyme used in this study is known to be able to catalyse the conversion of dihydroflavonols into flavonols, and the conversion of 2*S*-naringenin into kaempferol [43, 44]. It is therefore very likely to be responsible for producing kaempferol as a side product [12]. A different choice of source for the ANS enzyme may lead to a higher specificity of the microbial production system for anthocyanins, although all characterized ANS proteins have been observed to have this side activity [13]. ANS requires ferrous iron and 2-oxoglutarate for full activity. In vitro studies showed that ascorbic acid is also required or at least beneficial for activity of ANS [45, 46]. Therefore, supplementing the medium with ascorbic acid may improve ANS activity in vivo.

The performance of the glucosyltransferase 3GT in this work is still unclear. The production of kaempferol 3-*O*-glucoside is clearly observed, which confirms the presence of 3GT activity. In Table 3, the levels of kaempferol 3-*O*-glucoside are consistently equivalent to the levels of kaempferol, indicating satisfactory glucosyltransferase activity. On the other hand, pelargonidin 3-*O*-glucoside was in all cases much lower in concentration (if detectable at all) than its aglycone. In Arabidopsis, the 3GT (gene At5g17050) is known to play a crucial role in anthocyanin biosynthesis, since its deletion from the Arabidopsis genome results in strong reductions in anthocyanin accumulation in this plant [47]. Furthermore, in vitro enzyme assays have shown that this enzyme is able to mediate pelargonidin glycosylation [47]. An alternative explanation for the lack of P3G could be offered by the presence of glucosidases in yeast that specifically degrade pelargonidin 3-*O*-glucoside once it has been formed, as has been suggested by [24, 25]. However, deletion of the two enzymes that display the highest deglycosylation activity on P3G in in vitro assays did not result in increased pelargonidin 3-*O*-glucoside levels (Table 3). Possibly, other glucosidases exist in yeast that can also act on pelargonidin 3-*O*-glucoside, which have yet to be identified. Alternatively, 3GT enzymes with a higher specific activity on pelargonidin would need to be expressed in yeast. Wellmann et al. described that a 3GT from strawberry dramatically reduced the main reaction of ANS towards a catechin dimer when feeding the monomeric catechin in an in vitro enzyme assay [10]. Instead, the major product was the cyanidin 3-*O*-glucoside, while without the recombinant 3GT the respective aglycone cyanidin was only a minor product. Such a “pull” effect can, most probably, increase the flux towards the anthocyanin in yeast also.

Local concentrations of naringenin may also play a role in the limited production of pelargonidin. Though only a small fraction of de novo produced naringenin is finally converted into pelargonidin and pelargonidin 3-*O*-glucoside, production of these anthocyanin compounds may be increased by additional supplementation of naringenin to the culture. Also, boosting of de novo naringenin production, by preventing phloretic acid production through gene replacement of *TSC13* [30], indeed resulted in higher yields of pelargonidin (Table 3).

Anthocyanins, such as pelargonidin 3-*O*-glucoside and its aglycon, are distinguished from other polyphenols in that they are apparently not secreted into the medium. In previous work, compounds like naringenin and resveratrol could readily be recovered from the medium [7, 26]. Also, in the current work, dihydrokaempferol and kaempferol 3-*O*-glucoside were found in the medium. However, here, pelargonidin and pelargonidin 3-*O*-glucoside could not be detected in the medium, even when using sensitive LC–MS technology. Possibly, the positive charge of anthocyanins prevents their secretion through the secretion channels that export other polyphenols from yeast. Clearly, anthocyanin accumulation in the medium would be much preferred for easy recovery of the pigments from yeast cultures.

## Conclusions

Here we report that *S. cerevisiae* could be engineered for the de novo production of pelargonidin and pelargonidin 3-*O*-glucoside by expressing *PAL*, *TAL*, *C4H*, *CPR*, *4CL*, *CHS*, *CHI*, *F3H*, *DFR*, *ANS* and *3GT*. At the same time, a number of potential metabolic bottlenecks have been identified, ranging from enzyme kinetics and specificity, to anthocyanin export. Solving these will have a direct major impact on the efficiency of the system and are important targets for future attempts to promote yeast as a production system for anthocyanin pigments. Other plant pigments, such as carotenoids and betalains, have already been successfully produced in yeast [48, 49], and the results generated in this work represent an important framework for future development and sustainable production of this category of plant secondary metabolites.

## Methods

### Strains and maintenance

All *S. cerevisiae* strains used in this study (Table 4) are derived from strain IMK393 [7]. In general, yeast strains were grown on synthetic medium (6.7 g/L yeast nitrogen base without amino acids) with 20 g/L glucose (SMG) and appropriate growth factors to supplement the specific auxotrophic requirements of the strains (20 mg/L uracil, 20 mg/L histidine, 20 mg/L tryptophan and 120 mg/L leucine). Strains harbouring plasmids pCPW002, pCPW006 and pCPW007 were grown in the above medium supplemented with 1.5 mM naringenin (SMNar). Glycerol stocks were prepared by adding a final concentration of 20% glycerol to the culture and 1.5 mL aliquots were stored at − 80 °C.

**Table 4:** *Saccharomyces cerevisiae* strains used in this study

Name	Relevant genotype	Contains plasmid	Origin
IMK393	*MATalpha ura3*-*52 his3*-*Δ1 leu2*-*3112 trp1*-*289MAL2*-*8cSUC2 Δaro3::loxP ARO4*^*G226S*^ *pdc6Δ::loxP pdc5Δ::loxP aro10Δ::loxP*		[7]
IMX106	IMK393 *pyk2Δ*::*LEU2*-*TDH3*_*P*_-*atCHI1*-*CYC1*_*T*_ *TPI1p*-*atCHS3*-*ADH1*_*T*_*, TEF1*_*P*_-*at4CL3*-*TEF1*_*T*_	pUDE172	[7]
IMX106-F	IMX106	pF	This study
IMX106-FD	IMX106	pFD	This study
IMX106-FDA	IMX106	pFDA	This study
IMX106-FDA3	IMX106	pFDA3	This study
PATW002	IMK393	pCPW002	This study
PATW003	IMK393	p414-TEF1p-Cas9-CYC1t	This study
PATW004	PATW003 X-2::*TEF1*_*P*_-*At4CL3*-*TEF1*_*T*_ *TPI1*_*P*_-*AtCHS3*-*ADH1*_*T*_ *TDH3*_*P*_-*AtCHI1*-*CYC1*_*T*_	p414-TEF1p-Cas9-CYC1t	This study
PATW005	PATW004 XII-2::*TDH3*_*P*_-*AtPAL1*-*CYC1*_*T*_ *TPI1*_*P*_-*coC4H*-*ADH1*_*T*_ *PGI1*_*P*_-*coCPR1*-*PGI1*_*T*_ *TEF1*_*P*_-*coCHS3*-*TEF1*_*T*_	p414-TEF1p-Cas9-CYC1t	This study
PATW006	PATW005 *can1*Δ::*GAP1*_*P*_-*coF3H*-*TEF1*_*T*_ *TPI1*_*P*_-*coANS*-*PGK1*_*T*_ *TEF1*_*P*_-*coGhDFR*-*CYC1*_*T*_ *PGI1*_*P*_-*co3GT*-*ADH1*_*T*_	p414-TEF1p-Cas9-CYC1t	This study
PATW011	IMK393	pCPW006	This study
PATW012	IMK393	pCPW007	This study
PATW034	PATW006 *exg1Δ::TEF1*_*P*_-*coCHS3*-*TEF1*_*T*_	p414-TEF1p-Cas9-CYC1t	This study
PATW044	PATW034 *spr1Δ::TDH3*_*P*_-*coTAL1*-*CYC1*_*T*_	p414-TEF1p-Cas9-CYC1t	This study
PATW066	PATW005		This study
PATW072	PATW044 *tsc13*Δ::*TSC13*_*P*_-*coMdERC*-*TSC13*_*T*_	p414-TEF1p-Cas9-CYC1t	This study
PATW079	PATW006		This study
PATW080	PATW044		This study
PATW076	PATW072		This study

*E. coli* DH5α electrocompetent cells were used for bacterial transformations. For plasmid propagation, *E. coli* DH5α cells were cultured in LB medium supplemented with ampicillin (100 mg/L) at 37 °C with 250 rpm agitation. Solid medium was obtained by addition of 15 g/L agar.

### Media and cultivations

Shake flask cultures were grown in 250 mL baffled shake flasks with 50 mL SMG medium at 30 °C while shaking at 250 rpm. The shake flasks were inoculated to an OD_600_ of 0.2 with cells, resuspended in 2 mL of medium, obtained from a pre-culture grown in similar conditions. Samples of 1 mL were taken in time to follow growth and product formation. Optical density was measured at 600 nm using an Ultraspec 10 cell density meter (Amersham Biosciences).

Aerobic batch cultures were performed in 2-L bioreactors (Applikon, Delft, The Netherlands) using a working volume of 1.4 L. The bioreactors were filled with synthetic medium with extra nitrogen source (SMN). SMN contained 3 g/L KH_2_PO_4_, 0.5 g/L MgSO_4_·7H_2_O, and trace elements [50] and 10 g/L (NH_4_)_2_SO_4_ [7]. After heat sterilization of the bioreactors, 20 g/L glucose, 0.2 g/L antifoam C (Sigma-Aldrich, Zwijndrecht, The Netherlands), and filter-sterilized vitamins solution [50] were added. To sustain growth of the auxotrophic strains, the medium was further supplemented with a filter-sterilized mixture to a final concentration of 150 mg/L uracil, 500 mg/L leucine, 75 mg/L tryptophan, and 125 mg/L histidine [51, 52]. The bioreactors were inoculated from exponentially growing cells in pre-culture flasks containing 100 mL of SMN to an initial OD_660nm_ of 0.2. The aerobic batch cultivations were performed at 30 °C and at a stirrer speed of 800 rpm. The culture pH was maintained at 5.0 by automated addition of 2 M KOH and pressurized air was sparged through the bioreactors at 0.7 L/min.

### Plasmid and strain construction

All primers were supplied by IDT and are listed in a table (see Additional file 5). Preparative PCRs for cloning were performed using Q5 High-Fidelity DNA Polymerase (New England BioLabs). PCR conditions were adapted to the guidelines of the respective manufacturer. Restriction enzymes were obtained from Thermo Fisher Scientific and used according to the manufacturer’s instructions. PCR products were purified by using the NucleoSpin^®^ Gel and PCR Clean-up kit. Plasmids were isolated from *E. coli* DH5α overnight cultures using the NucleoSpin^®^ Plasmid EasyPure kit. DNA and plasmid isolation from *S. cerevisiae* was performed as previously described [53]. All yeast transformations were done using standard Lithium acetate methods according to [54].

The codon optimized genes of *A. thaliana F3H* (*coF3H*; Accession number: NP_190692), *ANS* (*coANS*; NP_194019), *UGT78D2* (*co3GT*; NP_197207), and *G. hybrida DFR* (*coGhDFR*; P51105) were ordered as synthetic genes (Genscript) as promoter, ORF terminator constructs. Codon optimization was performed using the Jcat algorithm [55]. For assembling them into plasmids, promoter-ORF-terminator fragments were amplified using Q5 polymerase (NEB) with primers that carried a restriction site at the 5′-end to facilitate cloning of the PCR products into vector pAT423 or p424_GPD (Table 5). The vectors pF, pD, p3GT, pFD, pFDA and pFDA3 were made by using in vivo homologous recombination in *S. cerevisiae* and by standard restriction ligation cloning (Table 5). Each construct was verified by DNA sequencing. Strain IMX106 was transformed with the different constructs pF, pFD, pFDA and pFDA3, resulting in strains IMX106-F, IMX106-FD, IMX106-FDA, IMX106-FDA3, respectively (Table 4).

**Table 5:** Plasmids used in this study

Name	Relevant characteristics	Origin
p414-TEF1p-Cas9-CYC1t	Centromeric plasmid, AmpR, *TRP1*, TEF1p-Cas9-CYC1t	[22]; Addgene#43802
p426-SNR52p-gRNA.CAN1.Y-SUP4t	2 μm ori, AmpR, *URA3*, gRNA-CAN1.Y	[22]; Addgene#43803
p426-SNR52p-gRNA.X-2.Y-SUP4t	2 μm ori, AmpR, *URA3*, gRNA-X-2.Y	This study
p426-SNR52p-gRNA.XII-2.Y-SUP4t	2 μm ori, AmpR, *URA3*, gRNA-XII-2.Y	This study
p426-SNR52p-gRNA.SPR1.Y-SUP4t	2 μm ori, AmpR, *URA3*, gRNA-SPR1.Y	This study
p426-SNR52p-gRNA.EXG1.Y-SUP4t	2 μm ori, AmpR, *URA3*, gRNA-EXG1.Y	This study
p426-SNR52p-gRNA.TSC13.Y-SUP4t	2 μm ori, AmpR, *URA3*, gRNA-TSC13.Y	This study
pESC-TRP	Template for A-*TRP*1-B	Agilent
p424_GPD	2 μm ori, AmpR, *TRP1*	[57]
pAT423	2 μm ori, AmpR, *HIS3*	[58]
pF	pAT423, *TDH*_*P*_-*coF3H*-*TDH*_*T*_	This study
pD	pAT423, *TDH*_*P*_-*coGhDFR*-*TDH*_*T*_	This study
p3GT	p424_GPD, *PGI1*_*P*_-*co3GT*-*CYC*_*T*_	This study
pFD	pAT423, *ADH*_*P*_-*coF3H*-*ADH*_*T*_, *TDH*_*P*_-*coGhDFR*-*TDH*_*T*_	This study
pFDA	pAT423, *GPD*_*P*_-*coF3H*-*TEF*_*T*_, *TDH*_*P*_-*coGhDFR*-*TDH*_*T*_, *ADH*_*P*_-*coANS*-*ADH*_*T*_	This study
pFDA3	pAT423, *GPD*_*P*_-*coF3H*-*TEF*_*T*_, *TDH*_*P*_-*coGhDFR*-*TDH*_*T*_, *ADH*_*P*_-*coANS*-*ADH*_*T*_, *PGI1*_*P*_-*co3GT*-*CYC*_*T*_	This study
pUC57 + AtF3H_BN_	Template for B-*GPD*_*P*_-*coF3H*-*TEF1*_*P*_-N	Synthetic construct
pUC57 + AtANS_NF_	Template for N-*TPI*_*P*_-*coANS*-*PGK*_*T*_-F	Synthetic construct
pUC57 + 2 µm_FG_	Template for F-2µm-G	Synthetic construct
pUC57 + GhDFR_GO_	Template for G-*TEF1*_*P*_-*coGhDFR*-*CYC1*_*P*_-O	Synthetic construct
pUC57 + MtDFR_GO_	Template for G-*TEF1*_*P*_-*coMtDFR*-*CYC1*_*P*_-O	Synthetic construct
pUC57 + AaDFR_GO_	Template for G-*TEF1*_*P*_-*coAaDFR*-*CYC1*_*P*_-O	Synthetic construct
pUC57 + At3GT_OI_	Template for O-*PGI1*_*P*_-*co3GT*-*ADH*_*T*_-I	Synthetic construct
pUDE172	Template for *TDH3*_*P*_-*atPAL1*-*CYC1*_*T*_, *TPI*_*P*_-*coC4H*-*ADH*_*T*_, *PGI*_*P*_-*coCPR1*-*PGI*_*T*_	[7]
pUDE188	Template for *TDH3*_*P*_-*coCHS3*-*CYC1*_*T*_, *TEF1*_*P*_-*coCHS3*-*TEF1*_*P*_, I-*E. coli*-A	[7]
pUDI065	Template for *TDH3*_*P*_-*atCHI1*-*CYC1*_*T*_, *TPI*_*P*_-*atCHS3*-*ADH*_*T*_, *TEF*_*P*_-*at4CL3*-*TEF*_*T*_	[7]
pUDI069	Template for *TDH3*_*P*_-*coTAL1*-*CYC1*_*T*_	[7]
pCPW002	2 μm ori, AmpR, *TRP1*, PGAP1-*AtF3H*-TTEF1, *TPI*_*P*_-*coANS*-*PGK*_*T*_, *TEF1*_*P*_-*coGhDFR*-*CYC1*_*P*_, *PGI1*_*P*_-*co3GT*-*ADH*_*T*_	This study
pCPW006	2 μm ori, AmpR, *TRP1*, PGAP1-*AtF3H*-TTEF1, *TPI*_*P*_-*coANS*-*PGK*_*T*_, *TEF1*_*P*_-*coMtDFR*-*CYC1*_*P*_, *PGI1*_*P*_-*co3GT*-*ADH*_*T*_	This study
pCPW007	2 μm ori, AmpR, *TRP1*, PGAP1-*AtF3H*-TTEF1, *TPI*_*P*_-*coANS*-*PGK*_*T*_, *TEF1*_*P*_-*coAaDFR*-*CYC1*_*P*_, *PGI1*_*P*_-*co3GT*-*ADH*_*T*_	This study

The episomal expression vectors pCPW002, pCPW006 and pCPW007 were assembled in vivo using 60 bp synthetic homologous recombination sequences [56]. The *M. truncatula DFR* (co*MtDFR*; XP_013466134) and *A. andreanum DFR* (*coAaDFR*; AAP20866) genes were ordered as yeast codon-optimized synthetic constructs. Gene fragments *coF3H*_BN_, *coANS*_NF_, 2 µm_FG_, *coGhDFR*_GO_, *coMtDFR*_GO_, *coAaDFR*_GO_, and co*3GT*_OI_ were synthesized by GenScript. The fragments *TRP*_AB_ and *E. coli*_IA_ were amplified from pESC-TRP to pUDE188, respectively. Correct assembly of the plasmids was confirmed via restriction enzyme analysis and sequencing (Macrogen). Strain IMK393 was transformed with the different constructs pCPW002, pCPW006 and pCPW007, resulting in strains PATW002, PATW011 and PATW012, respectively (Table 4).

Integration of gene fragments and knock-out of genes was obtained by using the p414-TEF1p-Cas9-CYC1t and p426-SNR52p-gRNA.CAN1.Y-SUP4t plasmids, as previously described [22]. The Yeastriction webtool was used to select for specific gRNA targeting sites in *S. cerevisiae* loci [59]. The gRNA Designer software of ATUM (https://www.atum.bio/eCommerce/cas9/input) was used to select for specific gRNA targeting sites in *S. cerevisiae* genomic regions. New gRNA target sequences were introduced in the template plasmid p426-SNR52p-gRNA.CAN1.Y-SUP4t by extension PCR. Primers containing the new gRNA target sequence were used in combination with primers ML009/ML010 in the 2 µm ori, creating two DNA fragments with minimally 60 bp overlaps. Both fragments were transformed to *S. cerevisiae* for in vivo assembly. The new gRNA plasmid was isolated from *S. cerevisiae* and transformed into *E. coli*. Plasmid was isolated from *E. coli* and correct replacement of the gRNA target sequence was confirmed via sequencing using primers ML017/018.

In general, for each new integration, an *S. cerevisiae* strain containing the p414-TEF1p-Cas9-CYC1t plasmid was transformed with a gRNA plasmid and integration fragment(s). Each integration fragment contained 60 bp overlaps upstream and downstream of the double strand break. In case of a gene knockout, at least 300 bp of the target gene sequence was deleted. Before each next integration, the gRNA plasmid was removed by growing the strain on liquid rich medium and plating on non-selective medium, subsequently, plasmid removal was confirmed by restreaking the same colony on selective and non-selective medium. Both gRNA and Cas9 plasmids were removed before strains were analysed in culturing experiments.

*Saccharomyces cerevisiae* strain IMK393 was transformed with p414-TEF1p-Cas9-CYC1t yielding strain PATW003. The expression cassette *at4CL3*-*atCHI1*-*atCHS3* for genomic integration was amplified using primers CP85/86 from plasmid pUDI065. Strain PATW003 was transformed with the gRNA.X-2 plasmid and the *at4CL3*-*atCHI1*-*atCHS3* integration fragment. Correct integration was verified by colony PCR and sequencing. After gRNA plasmid removal, this resulted in strain PATW004. The expression cassettes *atPAL1*-*coC4H*-*coCPR1* and *coCHS3* for genomic integration were amplified from plasmids pUDE172 and pUDE188 using, respectively, primers CP87/88 and CP89/90. Strain PATW004 was transformed with the gRNA.XII-2 plasmid and the *atPAL1*-*coC4H*-*coCPR1* and *coCHS3* integration fragments. Correct integration was verified by colony PCR and sequencing. After gRNA and Cas9 plasmid removal this resulted in strains PATW005 and PATW066, respectively.

The expression cassettes *coGhDFR*-*co3GT* and *coF3H*-*coANS* for genomic integration were amplified from plasmid pCPW002 using primers CP81/82 and CP83/84, respectively. Strain PATW005 was transformed with the gRNA.CAN1 plasmid and the *coGhDFR*-*co3GT* and *coF3H*-*coANS* integration fragments. Correct integration was verified by colony PCR and sequencing. After gRNA and Cas9 plasmid removal this resulted in strains PATW006 and PATW079, respectively.

The *EXG1* and *SPR1* knockouts were combined with introducing *coCHS3* and *coTAL1* (from *R. capsulatus*; WP_013066811). The expression cassette of *coCHS3* for genomic integration was amplified using primers CP166/167 from plasmid pUDE188. Strain PATW006 was transformed with the gRNA.EXG1 plasmid and the *coCHS3* integration fragment. Correct integration was verified by colony PCR and sequencing. After gRNA plasmid removal, this resulted in strain PATW034. The expression cassette *coTAL1* for genomic integration was amplified using primers CP168/169 from plasmid pUDI069. Strain PATW034 was transformed with the gRNA.SPR1 plasmid and the *coTAL1* integration fragment. Correct integration was verified by colony PCR and sequencing. After gRNA and Cas9 plasmid removal this resulted in strains PATW044 and PATW080, respectively.

The native open reading frame (ORF) of *TSC13* was replaced by its gene homologue from *M. domestica* (*MdECR*; accession XP_008382818), leaving the native TSC13 promoter and terminator intact. A yeast codon optimized *MdECR* gBlock gene fragment (*coMdECR*) was ordered from Integrated DNA Technologies. The *coMdECR* gene was amplified using primers CP182/183. Strain PATW044 was transformed with the gRNA.TSC13 plasmid and the *coMd*ECR integration fragment. Correct integration of *coMd*ECR was verified by colony PCR and sequencing. After gRNA and Cas9 plasmid removal this resulted in strains PATW072 and PATW076, respectively.

### Analytical methods

For product analysis of IMX106 strains, they were grown in 10 mL of synthetic dropout medium and incubated at 30 °C while shaking for 65 h. The cultures were mixed with 3 mL of acidified methanol (1% HCl) and frozen in liquid nitrogen. The cultures were lyophilized and the dried pellet was dissolved in 1 mL 80% methanol and 20 µL of the sample was analysed by HPLC. HPLC was carried out with a Nucleodur 100-5 C18 ec column on a 1298 Infinity HPLC System (Agilent Technologies). The mobile phase consisted of a gradient of solvent A (water with 0.1% *p*-phosphoric acid) and solvent B (acetonitrile with 0.1% phosphoric acid). The sample run was programmed as followed: gradient to 50% B for 25 min, isocratic elution of 50% B, gradient to 100% for 7 min and re-equilibration with 100% A for 5 min. The conversion of colourless leucopelargonidin to pelargonidin was achieved by acidification with 1% butanol/HCl and subsequent incubation for 5 min at 95 °C according to Reddy [60]. The resulting anthocyanidin was concentrated in an isoamyl alcohol phase for visualisation.

Optical density of shake flask experiments was measured at 600 nm using an Ultraspec 10 cell density meter (Amersham Biosciences). Glucose, ethanol, glycerol and acetate were analyzed by HPLC using an ICS5000 HPLC system (Thermo Scientific) equipped with a Dionex DP pump, Dionex AS-AP autosampler, Dionex VWD UV detector operated at 210 nm and Shodex RI detector operated at 35 °C. An Aminex HPX-87H cation-exchange column was used with a mobile phase of 0.16 N H_2_SO_4_ and was operated at 0.8 mL/min and 60 °C. 10 mM dimethylsulfoxide in 0.04 N H_2_SO_4_ was used as internal standard.

Optical density of bioreactor cultivations was measured by using a Jenway 7200 spectrophotometer (Jenway, Staffordshire, United Kingdom) at 660 nm. Cell dry weights were determined via filtration of 10 mL of well-mixed sample over dry nitrocellulose membrane filters with a pore size of 0.45 µm (Pall Corporation, Port Washington, NY). Filters were washed three times with demineralized water and dried in a microwave oven for 20 min at 360 W. For analysis of carbon dioxide production and oxygen consumption in the bioreactor, the off-gas was first cooled with a condenser (2 °C) and dried with a Permapure MD-110-48P-4 dryer (Perma pure, Lakewood, NJ), after which CO_2_ and O_2_ concentrations in the off-gas were measured with a NGA 2000 Rosemount gas analyser (Rosemount Analytical, Irvine, CA). Supernatant was obtained via centrifugation of the culture broth, which was subsequently measured via high-performance liquid chromatography (HPLC) on an Agilent 1260 Infinity (Agilent Technologies, Santa Clara, CA) fitted with an Aminex HPX-87H ion exchange column (Bio-Rad, Hercules, CA) operated at 60 °C with 5 mM H_2_SO_4_ as mobile phase at a flow rate of 0.6 mL/min. Detection was of extracellular metabolites was done by an Agilent 1260 refractive-index detector and an Agilent 1260 VWD detector. Corrections for ethanol evaporation were calculated according to [52].

For measurement of extracellular phenylpropanoic and flavonoid compounds in shake flask and bioreactor experiments, culture samples were diluted with an equal volume of 100% ethanol. After vigorous mixing, cells were centrifuged at maximum speed in a microcentrifuge for 10 min. The supernatant was analysed using HPLC. Caffeic acid in 100% ethanol was used as internal standard. For intracellular phenylpropanoic and flavonoid compounds, cell pellets were resuspended in 2 mL acidified methanol (0.75% HCl) and stored at − 80 °C for 24 h. Next, the samples were lyophilized for 24 h using an Edwards Modulyo freeze drier at − 40 °C. The dried pellet was resuspended in 2 mL acidified methanol (2% HCl) and stored overnight at − 80 °C. The cell pellet samples were centrifuged at maximum speed in a microcentrifuge at 4 °C for 10 min. The supernatant was analysed using HPLC.

For phenylpropanoic and flavonoid compounds, measurements were made using a Waters 2695 HPLC and UV absorbance was performed with a Waters 2996 photodiode array detector. Coumaric acid, phloretic acid, naringenin, dihydrokaempferol, kaempferol, kaempferol 3-*O*-glucoside, pelargonidin and pelargonidin 3-*O*-glucoside were measured, respectively at 312, 280, 280, 360, 360, 510 and 510 nm using a Luna C18(2) analytical column (2.0 × 150 mm, 100 Å, particle size 4 μm; Phenomenex) operating at 40 °C. Eluent A, ultra-pure water: formic acid (1000:1, v/v), and eluent B, acetonitrile: formic acid (1000:1, v/v) were used at a total flow rate of 0.19 mL/min. The gradient started at 5% B and increased linearly to 35% B over 45 min; afterward the column was washed with 75% B and equilibrated at 5% A for 15 min before the next injection. LC–MS analysis was performed as previously described [61].

Coumaric acid, caffeic acid, phloretic acid, naringenin, dihydrokaempferol, kaempferol 3-*O*-glucoside and pelargonidin standards were obtained from Sigma Aldrich (Zwijndrecht, The Netherlands). Kaempferol and pelargonidin 3-*O*-glucoside standards were obtained from, respectively Cayman Chemical (Ann Arbor, Michigan, USA) and Extrasynthese (Lyon, France).

## Additional files

**Additional file 1.** Detection of leucopelargonidin in *S. cerevisiae* strains IMX106-F and IMX106-FD. Leucopelargonidin is visible as a red-coloured band within the isoamyl alcohol phase after treatment with acidified butanol.

**Additional file 2.** Growth and production time courses of extracellular product formation in *S. cerevisiae* strains PATW002, PATW011, and PATW012 in shake flask cultures. The strains were grown in shake-flasks with 50 mL SMNar (1.5 mM naringenin) and the OD_600_ and extracellular metabolite concentration of dihydrokaempferol (DHK), kaempferol and kaempferol 3-*O*-glucoside (K3G) expressed in µM were measured by HPLC in supernatant of cultures in time.

**Additional file 3.** Growth and production time courses of extracellular product formation in *S. cerevisiae* strains PATW066, PATW079, PATW080 and PATW076 in shake flask cultures. The strains were grown in shake-flasks with 50 mL SMG and the OD_600_ and extracellular metabolite concentration of coumaric and phloretic acids, naringenin, dihydrokaempferol (DHK), kaempferol and kaempferol 3-*O*-glucoside (K3G) expressed in µM were measured by HPLC in supernatant of cultures in time.

**Additional file 4.** LC–MS chromatograms of cell pellet extracts of *S. cerevisiae* strains PATW076 and IMK393. The strains were grown in a bioreactor and biomass samples were taken after the reconsumption of ethanol. Pelargonidin and pelargonidin 3-*O*-glucoside (P3G) were found in the PATW076 sample. Chromatogram characteristics: (A) m/z range 433.111-433.115, (B) m/z range 271.055-271.065.

**Additional file 5.** Table with primers used in this study. Table with primers used in this study.

## Abbreviations

PAL: phenylalanine ammonia lyase
C4H: cinnamate 4-hydroxylase), CPR (cytochrome P450 reductase
4CL: 4-coumaric acid-CoA ligase), CHS (chalcone synthase
CHI: chalcone isomerase
F3H: flavanone 3-hydroxylase
ANS: anthocyanidin synthase
3GT: anthocyanin 3-*O*-glucosyltransferase
DFR: dihydroflavonol 4-reductase
TAL: tyrosine ammonia lyase
DHK: dihydrokaempferol
DHQ: dihydroquercetin
DHM: dihydromyricetin
P3G: pelargonidin 3-*O*-glucoside
C3G: cyanidin 3-*O*-glucoside
K3G: kaempferol 3-*O*-glucoside
