# Supplementary figures and images for: Engineering de novo anthocyanin production in Saccharomyces cerevisiae
Source: Microb Cell Fact. 2018 Jul 3;17:103. doi: 10.1186/s12934-018-0951-6 (PMC6029064; doi:10.1186/s12934-018-0951-6)

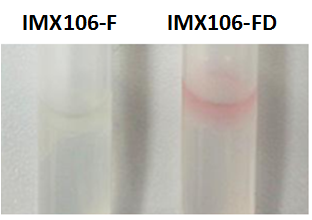

Supplement: Supplementary file 1 — Additional file 1. Detection of leucopelargonidin in S. cerevisiae strains IMX106-F and IMX106-FD. Leucopelargonidin is visible as a red-coloured band within the isoamyl alcohol phase after treatment with acidified butanol. [file 12934_2018_951_MOESM1_ESM.png]

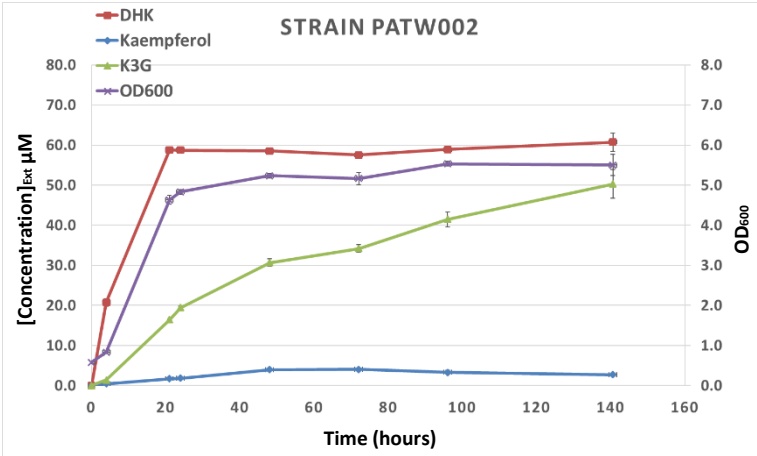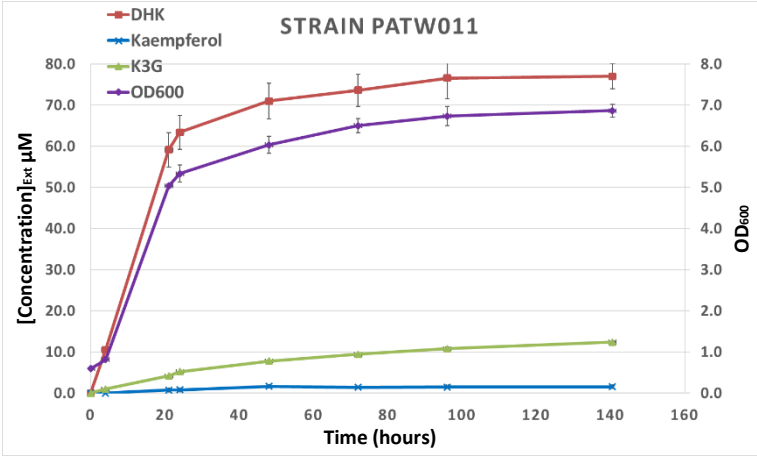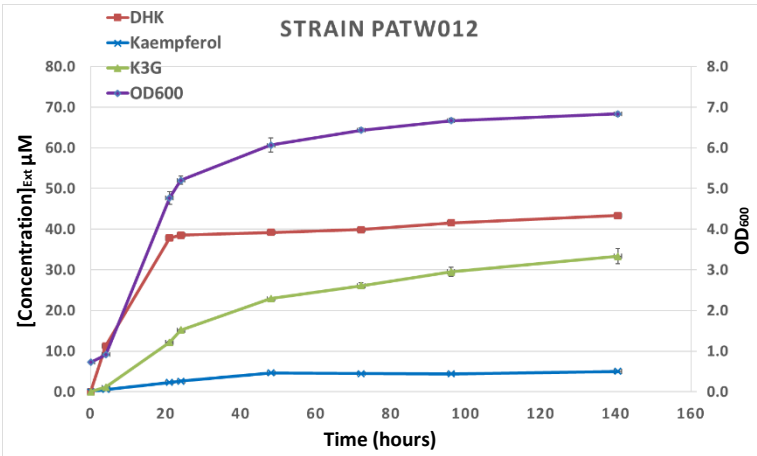

Supplement: Supplementary file 2 — Additional file 2. Growth and production time courses of extracellular product formation in S. cerevisiae strains PATW002, PATW011, and PATW012 in shake flask cultures. The strains were grown in shake-flasks with 50 mL SMNar (1.5 mM naringenin) and the OD600 and extracellular metabolite concentration of dihydrokaempferol (DHK), kaempferol and kaempferol 3-O-glucoside (K3G) expressed in µM were measured by HPLC in supernatant of cultures in time. [file 12934_2018_951_MOESM2_ESM.pdf]

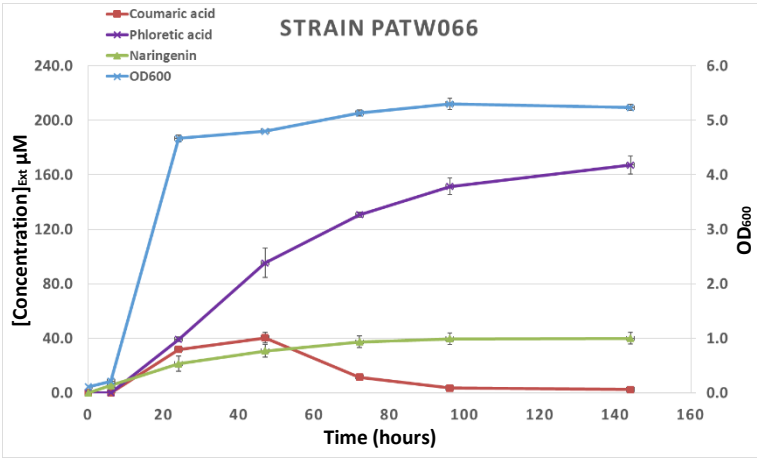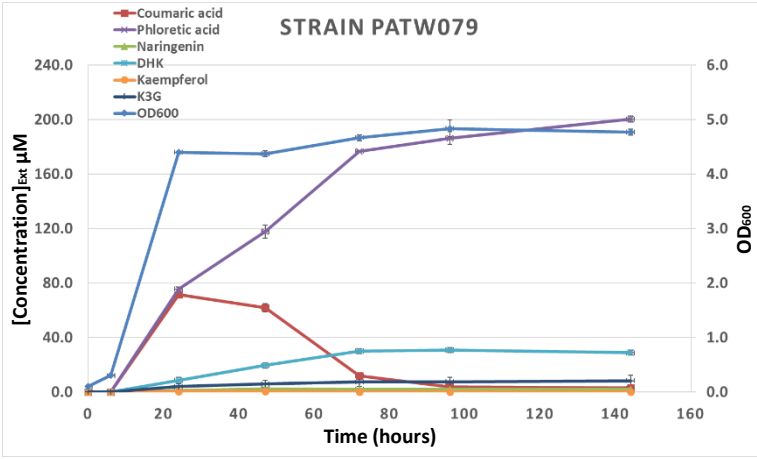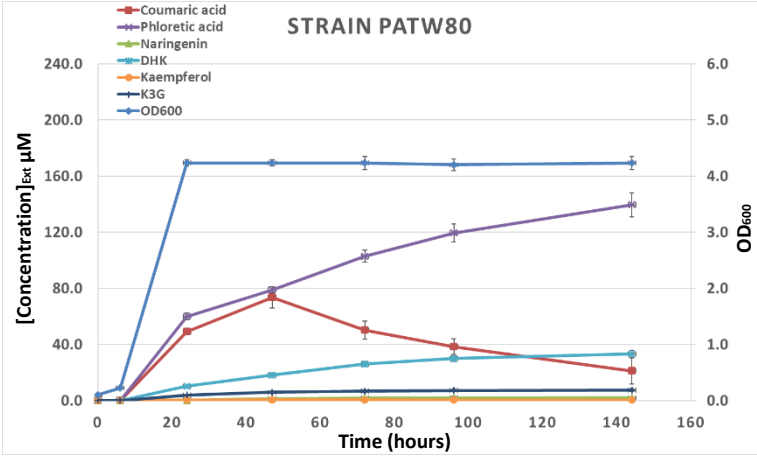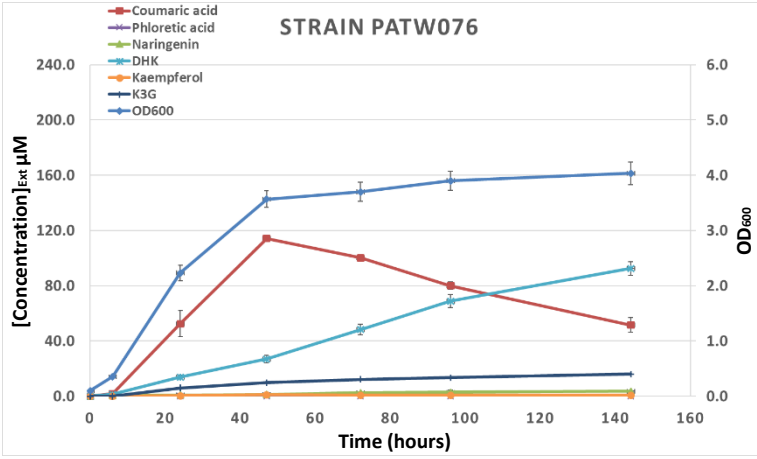

Supplement: Supplementary file 3 — Additional file 3. Growth and production time courses of extracellular product formation in S. cerevisiae strains PATW066, PATW079, PATW080 and PATW076 in shake flask cultures. The strains were grown in shake-flasks with 50 mL SMG and the OD600 and extracellular metabolite concentration of coumaric and phloretic acids, naringenin, dihydrokaempferol (DHK), kaempferol and kaempferol 3-O-glucoside (K3G) expressed in µM were measured by HPLC in supernatant of cultures in time. [file 12934_2018_951_MOESM3_ESM.pdf]

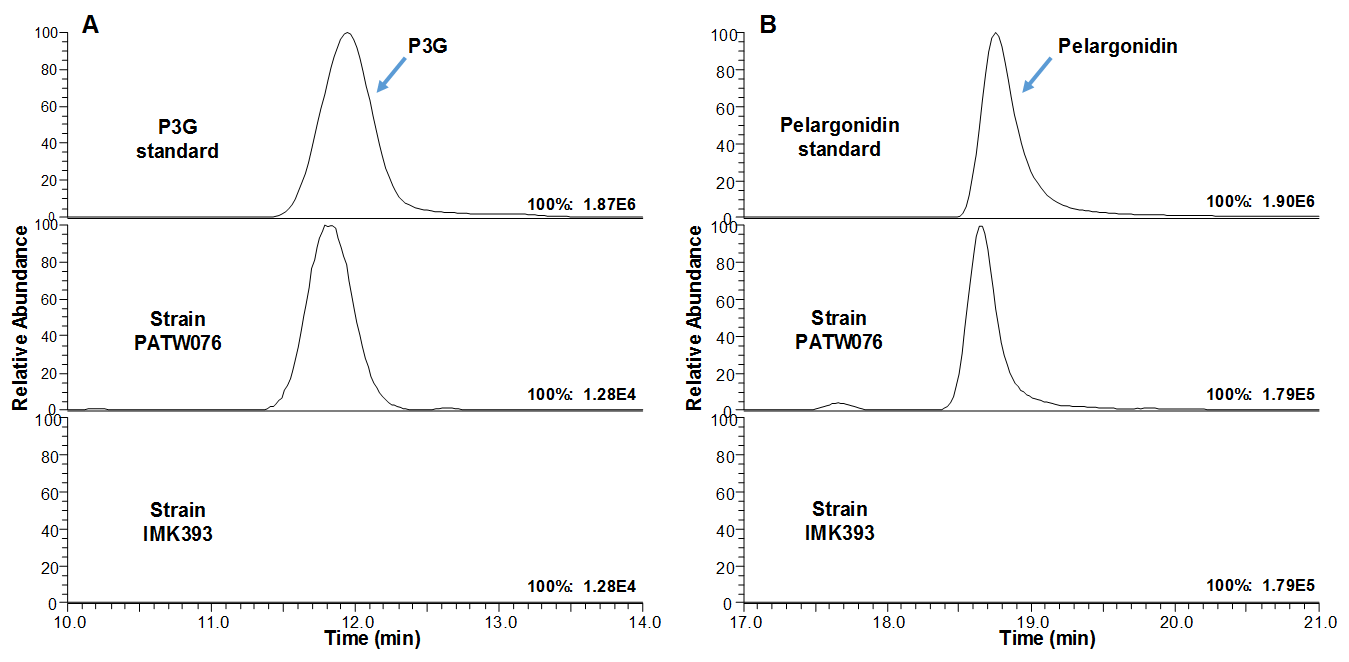

Supplement: Supplementary file 4 — Additional file 4. LC–MS chromatograms of cell pellet extracts of S. cerevisiae strains PATW076 and IMK393. The strains were grown in a bioreactor and biomass samples were taken after the reconsumption of ethanol. Pelargonidin and pelargonidin 3-O-glucoside (P3G) were found in the PATW076 sample. Chromatogram characteristics: (A) m/z range 433.111-433.115, (B) m/z range 271.055-271.065. [file 12934_2018_951_MOESM4_ESM.png]
