# Supplementary material for: Engineering de novo anthocyanin production in Saccharomyces cerevisiae
Source: Microb Cell Fact. 2018 Jul 3;17:103. doi: 10.1186/s12934-018-0951-6 (PMC6029064; doi:10.1186/s12934-018-0951-6)
Supplement: Supplementary file 5 — Additional file 5. Table with primers used in this study. Table with primers used in this study. [file 12934_2018_951_MOESM5_ESM.pdf]

Additional file 3

Name	Sequence	Description
**Assembly of pF, pFD, pFDA, and pFDA3**		
SH1	AAAACCTAGGATGGTTGCTGTTGAAAGAGTTG	AtANS Adh1-cassette FW
SH2	AAAAGGCCGGCCTTAGTCGTTCTTTTCAGAAAC	AtANS Adh1-cassette RV
SH3	AAAAGTCGACATGGAAGAAGACTCTCCAGC	GhDFR Tdh3-cassette FW
SH4	AAAAGCGGCCGCCTAATTACATGATTGACCTTC	GhDFR Tdh3-cassette RV
SH5	AAAACCTAGGATGGCTCCAGGTACTTTGAC	GhDFR Adh1-cassette FW
SH6	AAAAGGCCGGCCTTAAGCGAAGATTTGGTCAACTG	GhDFR Adh1-cassette RV
SH7	TACGACTCACTATAGGGCGAATTGGGTACCGGGCCCCCCCTCGAGGATTTCTTCTAGGCTTTGATGCAAGGTCC	AtF3H HR to pD FW
SH8	GATGAATCTCGGTGTGTATTTTATGTCCTCAGAGGACAACTCGACCAGTTTATCATTATCAATACTCGCC	AtF3H HR to pD RV
SH9	AAAGTTTACAAATGAATTTTTTCCGCCAGGATTTAATTAAGAGCTGGAATGATCGTTCAGCGCGTTCTCGG	At3GT HR to pFDA FW
SH10	CCAAGCGCGCAATTAACCCTCACTAAAGGGAACAAAAGCTGGAGCTCGCCTACGGTTCCCGAAGTATGCTGCTG	At3GT HR to pFDA RV
SH11	ATTATACTAGTATGACTAAGCCATCTGACCCAAC	At3GT to p426-GPD FW
SH12	ATAAACTCGAGTTAGATGATGTTAACAACAGC	At3GT to p426-GPD RV
**Assembly of pCPW002,**		
CP49	ACTATATGTGAAGGCATGGCTATGGCACGGCAGACATTCCGCCAGATCATCAATAGGCACGCGTTTCGGTGATGACGGTGAAAACCTC	A-TRP1-B FW
CP50	GTTGAACATTCTTAGGCTGGTCGAATCATTTAGACACGGGCATCGTCCTCTCGAAAGGTGCTGGAACAACACTCAACCCTATCTC	A-TRP1-B RV
CP43	CACCTTTCGAGAGGACGATG	B-AtF3H-N FW
CP44	TTCTAGGCTTTGATGCAAGGTC	B-AtF3H-N RV
CP45	GATCAGCAGCCACGATTGAGTC	N-AtANS-F FW
CP46	TGCCGAACTTTCCCTGTATGAAG	N-AtANS-F RV
CP47	CATACGTTGAAACTACGGCAAAGGATTG	F-2µm-G FW
CP48	AAGGGCCATGACCACCTGAT	F-2µm-G RV
CP93	GCCAGAGGTATAGACATAGCCAGACC	G-GhDFR-O FW
CP94	ATACTCCCTGCACAGATGAGTCAAG	G-ghDFR-O RV
ML108	GTGCCTATTGATGATCTGGCGGAATGTCTGCCGTGCCATAGCCATGCCTTCACATATAGTTGCGCGGAACCCCTATTTG	A-E.coli-I FW
ML109	TATTCACGTAGACGGATAGGTATAGCCAGACATCAGCAGCATACTTCGGGAACCGTAGGCGAGAGGCGGTTTGCGTATTGG	A-E.coli-I RV
**Assembly of pCPW006 and pCPW007**		
CP129	GTCAAGACAACGGTCACTAATCATGTAATTAGTTATGTCACGCTTACATTCACG	AaDFR-CYCt FW
CP130	CGTGAATGTAAGCGTGACATAACTAATTACATGATTAGTGACCGTTGTCTTGAC	CYCt-AaDFR RV
CP131	GAAAGCATAGCAATCTAATCTAAGTTTATGATGCACAAGGGTACTG	TEFp-AaDFR FW
CP132	CAGTACCCTTGTGCATCATAAACTTAGATTAGATTGCTATGCTTTC	AaDFR-TEFp RV
CP133	GTTCGTTAAGTCTACTAACAAGTAATCATGTAATTAGTTATGTCACGCTTACATTCACG	MtDFR1-CYCt FW
CP134	CGTGAATGTAAGCGTGACATAACTAATTACATGATTACTTGTTAGTAGACTTAACGAAC	CYCt-MtDFR1 RV
CP135	GAAAGCATAGCAATCTAATCTAAGTTTATGGGTTCTATGGCTGAAAC	TEFp-MtDFR1 FW
CP136	GTTTCAGCCATAGAACCCATAAACTTAGATTAGATTGCTATGCTTTC	MtDFR1-TEFp RV
**Replace p426 GTS**		
ML009	TCGGTATAGAATATAATCGGGGATGCC	2µm internal FW
ML010	GCGTTTACTGATTACTAGCGAAGCTG	2µm internal RV
ML017	TGTAAAACGACGGCCAGT	M13 FW
ML018	TCACACAGGAAACAGCTATGAC	M13 RV

**X-2::(At4CL3, AtCHS3, AtCHI1) integration**		
CP95	GATCTAGGAAGAGAGTAGTGGGCGGTTTTAGAGCTAGAAATAGC	X-2 gRNA GTS FW
CP96	CTAAAACCGCCCACTACTCTCTTCCTAGATCATTTATCTTTCACTGC	X-2 gRNA GTS RV
CP85	GTTAATTGTCGCTGTTATTGTCTAGATTTTTTCTCGGAGATGGCGCATCTATTTGCCGTCCAGTATAGCGACCAGCATTC	4CL3 CHS3 CHI1 repair FW
CP86	CATTCGAAGGTTAGGTGTGGCACAAAGCCGATAAGAAAAAGAAAAGTAGTGAGGACAGGCGCAAATTAAAGCCTTCGAGC	4CL3 CHS3 CHI1 repair RV
CP109	GCTCGATCTTCTATCCTCTTTAG	X-2_int diagnosis FW
CP110	CCATGTAAGAACGGAATAAAACAG	X-2_int diagnosis RV
CP51	ACGGCGAAACACACCTAATC	X-2_int diagnosis FW
CP55	ATGTCTTCATCCAACGCCTG	X-2_int diagnosis RV
**XII-2::(coAtPAL1, coAtC4H, coAtCPR1, coAtCHS3) integration**		
CP115	ATAAATGATCTTTGCTTTGTTCTGGTCCCTGTTTTAGAGCTAGAAATAGCAAG	XII-2 gRNA GTS FW
CP116	GCTCTAAAACAGGGACCAGAACAAAGCAAAGATCATTTATCTTTCACTGCG	XII-2 gRNA GTS RV
CP87	CTGGAAAGGCCTAAGTAATGTCGTAAACGCATTCTATCTGTACTTCAACTCTCCTCTGTGTCATTATCAATACTCGCCAT	PAL1 C4H CPR1 repair FW
CP88	GGAAGAGTAAAAAAGGAGTAGAAACATTTTGAAGCTATGGCGGAATTGAGCGCAAGTTC	PAL1 C4H CPR1 repair FW
CP89	CAACTGTTTAAAAGGATCATGAACTTGCGCTCAATTCCGCCATAGCTTCAAAATGTTTC	CHS3 repair FW
CP90	GTTCGAGAATGGAGAACTTCGTAGTCGGTTAAAACAGCTGTAGTGTTCTGGCCTTCTTAGCAGTATAGCGACCAGCATTC	CHS3 repair RV
CP111	CATATTTTGTCTTTTCGCGCC	XII-2_int diagnosis FW
CP112	CCTGTTTGGTAAATGAAGGCTAG	XII-2_int diagnosis RV
CP69	ATGACTTCTGCTTTGTACGC	XII-2_int diagnosis FW
CP32	AGTATGCTGTGCTTGGGTG	XII-2_int diagnosis RV
CP100	CGGTCTTCAATTTCTCAAGTTTCAG	XII-2_int diagnosis FW
CP102	CTCCTTCCTTTTCGGTTAGAGC	XII-2_int diagnosis RV
**CAN1::(coAtF3H, coAtANS, coGhDFR, coAt3GT) integration**		
CP81	ATGACAAATTCAAAAGAAGACGCCGACATAGAGGAGAAGCATATGTACAATGAGCCGGTCCATAGCTTCAAAATGTTTCTAC	DFR 3GT repair FW
CP82	GAATCATTTAGACACGGGCATCGTCCTCTCGAAAGGTGGCCTACGGTTCCCGAAGTATG	DFR 3GT repair RV
CP83	ATAGCCAGACATCAGCAGCATACTTCGGGAACCGTAGGCCACCTTTCGAGAGGACGATG	F3H ANS repair FW
CP84	GAGGATGTAACAGGGATGAATGTAGCCATTTCACCCAAGGACTGCGTGACAGAATATGCCAACGCAGAATTTTCGAGTTA	F3H ANS repair FW
CP146	CTTCAGACTTCTTAACTCCTG	CAN1_int diagnosis FW
CP103	CATAACCAACCAAGAACCGATG	CAN1_int diagnosis RV
CP35	GGTCAGGTTGCTTTCTCAGG	CAN1_int diagnosis FW
CP26	CCTATTTTGGGCATGTACGG	CAN1_int diagnosis RV
CP108	CCACTTGGCTTACCCAGAAG	CAN1_int diagnosis FW
CP147	CTTTGTGAGAACACTGTG	CAN1_int diagnosis RV
**EXG1::coCHS3 integration**		
CP176	TGATCCTTCTGTCAATATTTAGGTAGTTTTAGAGCTAGAAATAGCAAGTTAAAATAAGGC	EXG1 gRNA GTS FW
CP177	CTAGCTCTAAAACTACCTAAATATTGACAGAAGGATCATTTATCTTTCACTGCGGAGAAG	EXG1 gRNA GTS RV
CP191	GGTGGTTGGTTACTTCTTGAACCATACATTACTCCATCTTTGTTCGAGGCTTTCCGTACACATAGCTTCAAAATGTTTCTACTCC	coAtCHS3 repair FW
CP167	GTCTAGAACAGGACCCAATGGCTCATTAATCAATTCGATACCAATAACAGTGTCCAAGTACAGTATAGCGACCAGCATTC	coAtCHS3 repair RV
CP164	CGTTACTGTGTACGTTGTTGACTG	EXG1_int diagnosis FW
CP165	CAAACAGTCCAGTGGGATTCATTC	EXG1_int diagnosis RV
CP188	GAGACACATGCACTTGACTGAAG	EXG1_int diagnosis FW
CP189	CACAAGCAGAAGACATGTTACCG	EXG1_int diagnosis RV

**SPR1::coRcTAL1 integration**		
CP178	TGATCGGAAATATGGTTTGAAAGTAGTTTTAGAGCTAGAAATAGCAAGTTAAAATAAGGC	SPR1 gRNA GTS FW
CP179	CTAGCTCTAAAACTACTTTCAAACCATATTTCCGATCATTTATCTTTCACTGCGGAGAAG	SPR1 gRNA GTS RV
CP190	ACACCTTCTTTATTCGAGACTTTCCGTACTAATCCGTACAACGATGACGGTATTCCTGTTTGTAAAACGACGGCCAGT	coRcTAL1 repair FW
CP169	CTTCAAAAGCAAATTTTTCAATCTTTCCATGTCAATAACTGGACCTAACGGTTCATTGAGGCAAATTAAAGCCTTCGAGC	coRcTAL1 repair RV
CP162	GGTGGGTGGCTAGTATTGGAG	SPR1_int diagnosis FW
CP163	GATGGTCAATTATGACGCCATATTCG	SPR1_int diagnosis RV
CP174	ATTAATGGAAGTTTTGAGTGGTCATG	SPR1_int diagnosis FW
CP175	CGTCTTGTGCAGGATGATC	SPR1_int diagnosis RV
**TSC13::MdECR integration**		
CP180	TGATCTGAAAATACCTTGATTCAATGTTTTAGAGCTAGAAATAGCAAGTTAAAATAAGGC	TSC13 gRNA GTS FW
CP181	CTAGCTCTAAAACATTGAATCAAGGTATTTTCAGATCATTTATCTTTCACTGCGGAGAAG	TSC13 gRNA GTS RV
CP182	GCTATCTAGAAACCAATTGAGCTATTTGAGAGAGATACATATTTTGAATTTAATTTGAAAATGAAGGTTACTGTTGTTTC	coMdECR repair FW
CP183	CCACTTCGTGAAAGCTAATATCTCTTTACCTTGCATTTGGGCATGTTGCAAACAGGAGGATTACAAGAATGGTGGCAAG	coMdECR repair RV
CP184	TGAAAAGGGACTAAGAGCGTG	TSC13_int diagnostic FW
CP185	GATGAAAGCACCGAAAGACC	TSC13_int diagnostic RV
CP186	GACTTTGCCAGTTCAACCAGG	TSC13_int diagnostic FW
CP187	TGCTACTACGCCACTTCGTG	TSC13_int diagnostic RV
